# Final Results From a Large, Non‐Interventional, Phase 4 Study of Ruxolitinib for the Treatment of Myelofibrosis in Clinical Routine

**DOI:** 10.1111/ejh.70005

**Published:** 2025-07-06

**Authors:** Steffen Koschmieder, Susanne Isfort, Clemens Schulte, Lutz Jacobasch, Thomas Geer, Marcel Reiser, Michael Koenigsmann, Bernhard Heinrich, Jürgen Wehmeyer, Eyck von der Heyde, Hans Tesch, Benedikt Gröschl, Petra Bachhuber, Susanne Großer, Michael Koehler, Heike L. Pahl

**Affiliations:** ^1^ Department of Hematology, Oncology, Hemostaseology, and Stem Cell Transplantation, Faculty of Medicine RWTH Aachen University Aachen Germany; ^2^ Center for Integrated Oncology Aachen Bonn Cologne Düsseldorf (CIO ABCD) Aachen Germany; ^3^ Department of Hematology, Hemostasis, Oncology and Stem Cell Transplantation, Comprehensive Cancer Center Hannover Medical School Hannover Hannover Germany; ^4^ Gemeinschaftspraxis für Hämatologie und Onkologie Dortmund Germany; ^5^ Gemeinschaftspraxis Hämatologie—Onkologie Dresden Germany; ^6^ Medizinische Klinik III, Diakonie‐Klinikum Schwäbisch Hall Schwäbisch Hall Germany; ^7^ Praxis Internistischer Onkologie und Hämatologie Köln Germany; ^8^ Onkologisches Ambulanzzentrum (OAZ) Hannover Hannover Germany; ^9^ Hämatologie‐Onkologie im Zentrum MVZ GmbH Augsburg Germany; ^10^ Gemeinschaftspraxis für Hämatologie und Onkologie Münster Germany; ^11^ Onkologische Schwerpunktpraxis Dres. Ingo Zander und Eyck von der Heyde Hannover Germany; ^12^ Onkologische Gemeinschaftspraxis am Bethanien‐Krankenhaus Frankfurt/Main Germany; ^13^ Providing services for Novartis Pharma GmbH Nuremberg Germany; ^14^ Novartis Pharma GmbH Nuremberg Germany; ^15^ Specialty Practice for Psycho‐Oncology Magdeburg Germany; ^16^ Department of Hematology and Oncology, Medical Center Otto von Guericke University Magdeburg Germany; ^17^ Department of Medicine I, Medical Center – University of Freiburg, Faculty of Medicine University of Freiburg Freiburg Germany

**Keywords:** clinical routine, myelofibrosis, real‐life analysis, ruxolitinib

## Abstract

JAKoMo was a long‐term, multicenter, non‐interventional study observing the efficacy, safety, and quality of life (QOL) effects of ruxolitinib (RUX), managed per clinical routine at investigator discretion, for treatment of 943 patients with myelofibrosis (MF) in 122 German centers. Patients ≥ 18 years with a diagnosis of PMF or PPV‐MF or PET‐MF, who were suitable for in‐label treatment with RUX were eligible and could be included either before (479 previously RUX‐naïve [Arm A]) or after the start of treatment (464 RUX‐experienced patients [Arm B]) and were followed over 36 months. Arm A showed rapid (≤ 6 months), sustained improvements from baseline in all efficacy outcomes and most QOL measures. Both arms showed an ~17% increase in the proportion of patients experiencing a normal German QOL during follow‐up. Arm B entered the study with better outcomes and QOL than Arm A, with outcomes generally remaining stable over time. Adverse events were less common than in registrational trials, possibly due, in part, to lower real‐world RUX dosing. Survival was comparable to published data. The JAKoMo study demonstrates that real‐world RUX treatment of MF promotes significant and sustained clinical and QOL benefits, including improvement of general health and alleviation of MF‐associated fatigue. Maximum sustained responses were generally achieved within 6 months and associated with fewer adverse events than in published randomized trials, which may reflect more conservative and personalized real‐world dosing.

**Trial Registration:** The JAKOMO trial: http://clinicaltrials.gov/show/NCT05044026

## 1 Introduction

Myelofibrosis (MF) is a rare Philadelphia chromosome–negative myeloproliferative neoplasm (MPN) that presents either as a primary disease (PMF) or after progression of polycythemia vera (PPV‐MF) or essential thrombocythemia (PET‐MF). MF‐related symptoms are known to severely decrease patient quality of life (QOL), particularly with respect to fatigue, loss of vitality, and emotional burden accruing from depression or feelings of anxiety [1], and also from abdominal discomfort and sexual dysfunction [2]. Causes of death in patients with MF include progression to acute leukemia, infections, and cardiovascular events [3].

The pharmacologic management of MF has significantly improved due to the development of small‐molecule Janus kinase (JAK) inhibitors (JAKis). Ruxolitinib (RUX) is a JAK1/JAK2 inhibitor introduced in 2011/2012 as the first JAKi MF treatment. In pivotal Phase 3 Trials (COMFORT‐I and ‐II) [4, 5], it induced significant reductions in splenomegaly and symptomatic burden, which were confirmed in a global expanded access trial with broader eligibility criteria (JUMP) [6]. Moreover, RUX has shown a survival benefit in pooled data from the COMFORT studies [7], although these data must be viewed with caution as these studies were not powered for survival analysis.

As with all formal interventional trials, stratification introduced by the patient eligibility criteria for COMFORT and JUMP limits the generalizability of the results to routine clinical practice post‐approval. We have previously described interim data from a large, representative, non‐interventional, two‐arm, prospective study of RUX administered in the routine clinical setting to German patients with MF with or without prior JAKi experience (the JAKoMo study), in an interim assessment [8], on the JAKi‐naïve patient set only (Arm A). Here, we describe the final study analysis of all 943 JAKoMo patients from both the JAKi‐naïve and ‐experienced study arms.

## 2 Methods

### 2.1 Study Design

JAKoMo is a two‐arm, open‐label, Phase 4, non‐interventional study of patients with MF who were either JAKi naïve or pretreated with RUX. Patients ≥ 18 years with a diagnosis of PMF, according to the World Health Organization classification, or PPV‐MF or PET‐MF, according to the International Working Group for Myeloproliferative Neoplasms Research and Treatment (IWG‐MRT) criteria [9, 10], who were suitable for in‐label treatment with RUX were eligible. Patients in Arm A were JAK inhibitor treatment naïve when they entered the trial. In Arm B patients could be included after they had started RUX treatment. There were no further criteria.

Between September 2012 and September 2019, 928 patients (Arm A: *n* = 464 JAKi‐naïve patients; Arm B: *n* = 464 JAKi‐pretreated patients) eligible for analysis were enrolled across 122 centers in Germany.

Starting doses of RUX were based on platelet counts according to the SmPC. Dose reductions or interruptions were also recommended according to the SmPC. Pts were observed for up to 36 months after enrollment, unless discontinuation criteria according to the SmPC were met.

Adverse events (AEs) and concomitant diseases were coded according to the Medical Dictionary for Regulatory Activities (MedDRA) version 23.1.

The study was sponsored by Novartis Pharma GmbH (Novartis) and designed by Novartis in collaboration with S. Koschmieder as the medical leading investigator. The study was approved by the institutional review boards of the respective institutions (leading ethics committee: Ethics Committee at the Faculty of Medicine, RWTH Aachen University, Aachen, Germany) before enrollment of pts. and was conducted in accordance with the principles of the Declaration of Helsinki. The trial is registered with ClinicalTrials.gov (NCT05044026).

All patients provided written informed consent.

### 2.2 Statistics

The study analysis used epidemiologic methods with primary use of descriptive statistical methods. All data were analyzed descriptively. In cases of confidence intervals or *p* values, the analyses were also descriptive, therefore, no *α* adjustment for multiple tests was performed.

Data collection for this non‐interventional study started in 2012 and ended in 2019. Thus, RR6 was not documented in this study, but was calculated “post hoc” from existing data. As much more spleen length data is available from sonographic measurements, we decided to use these data for RR6 calculation. For a test of robustness of this method, we estimated the congruency of palpation and sonography data and found a good correlation, with a slight underestimation of spleen length as measured by palpation.

More detailed information on the JAKoMo trial (NCT05044026) can be found in the former publication [8].

#### 2.2.1 Statistical Details

The full analysis set (FAS) comprised all patients with documented informed consent, a diagnosis of PMF, PPV‐MF, or PET‐MF who had completed at least one documented post‐baseline (BL) visit. Study visit times were defined relative to the BL visit and were timed separately for Arm A and Arm B. All analyses were descriptive. Since monitoring was limited and thereby relevant endpoint data (e.g., on spleen response) was missing for certain timepoints, there could be a detection bias in our analysis. Summary statistics were employed, and statistical comparisons were unpowered and unadjusted for multiplicity. For further details on statistics please see Supporting Information Part S1.

## 3 Results

### 3.1 Patient Characteristics and Disposition

Between September 2012 and September 2019, a total of 1012 patients were enrolled at 122 clinical sites in Germany, with each site enrolling between 1 and 32 patients (median number of patients included per center was 7, interquartile range [IQR] 3–10). Sixty‐nine patients were subsequently excluded from analysis due to lack of data, leaving a total population of 943 that was divided into a previously RUX‐naïve arm (Arm A, 479 patients) and a RUX‐experienced arm (Arm B, 464 patients). A disposition diagram showing enrollment and patient flow through the study is depicted in Figure S1.

Patient BL characteristics and demographics are shown in Table 1. The study enrolled roughly equal numbers of males and females, with a mean age of ~70 years and broadly similar characteristics across the three MF categories. Overall, 60% of patients had available International Prognostic Scoring System (IPSS) risk data, either documented by the responsible physician or subsequently calculated from available data at the BL visit. This lower percentage of IPSS documentation was mostly caused by lack of 100% monitoring but may also reflect less estimation of IPSS relevance for real‐world management of MF patients.

**TABLE 1:** Demographics and baseline characteristics by study arm and MF diagnosis.

Except where otherwise stated, data are *n* (%)	Arm A (*n* = 479)				Arm B (*n* = 464)				All patients (*N* = 943)			
	PMF (*n* = 325)	PPV‐MF (*n* = 99)	PET‐MF (*n* = 55)	All Arm A (*n* = 479)	PMF (*n* = 318)	PPV‐MF (*n* = 99)	PET‐MF (*n* = 47)	All Arm B (*n* = 464)	PMF (*n* = 643)	PPV‐MF (*n* = 198)	PET‐MF (*n* = 102)	All patients (*N* = 943)
Age, mean (SD), [range], years	70.5 (10.3) [38–95]	71.1 (10.5) [32–93]	67.4 (12.9) [35–86]	70.2 (10.7) [32–95]	70.4 (11.7) [23–92]	70.1 (10.1) [42–88]	70.1 (11.9) [32–90]	70.3 (11.4) [23–92]	70.4 (11.0) [23–95]	70.6 (10.3) [32–93]	68.6 (12.4) [32–90]	70.3 (11.0) [23–95]
Sex												
Male	191 (58.8)	44 (44.4)	19 (34.5)	254 (53.0)	184 (57.9)	52 (52.5)	17 (36.2)	253 (54.5)	375 (58.3)	96 (48.5)	36 (35.3)	507 (53.8)
Female	134 (41.2)	55 (55.6)	36 (65.5)	225 (47.0)	134 (42.1)	47 (47.5)	30 (63.8)	211 (45.5)	268 (41.7)	102 (51.5)	66 (64.7)	436 (46.2)
BMI, mean (SD), [range], kg/m^2^ ^a^	25.5 (4.4) [16.0–45.0]	24.7 (4.3) [17.0–36.3]	23.4 (3.7) [18.5–39.1]	25.1 (4.3) [16.0–45.0]	25.9 (4.4) [15.6–45.5]	25.2 (4.2) [18.6–39.1]	24.9 (4.9) [14.5–34.9]	25.6 (4.4) [14.5–45.5]	25.6 (4.4) [15.6–45.5]	24.9 (4.2) [17.0–39.1]	24.1 (4.3) [14.5–39.1]	25.3 (4.4) [14.5–45.5]
Prior RUX treatment, median (IQR), [range], days	ND	ND	ND	0	ND	ND	ND	117 (65–274) [16–2366]	ND	ND	ND	ND
Smoking status												
*n* with data	310	94	54	458	297	95	46	438	607	189	100	896
No	232 (74.8)	76 (80.9)	41 (75.9)	349 (76.2)	234 (78.8)	77 (81.1)	35 (76.1)	346 (79.0)	466 (76.8)	153 (81.0)	76 (76.0)	695 (77.6)
Yes	30 (9.7)	5 (5.3)	5 (9.3)	40 (8.7)	20 (6.7)	6 (6.3)	5 (10.9)	31 (7.1)	50 (8.2)	11 (5.8)	10 (10.0)	71 (7.9)
Ex‐smoker	48 (15.5)	13 (13.8)	8 (14.8)	69 (15.1)	43 (14.5)	12 (12.6)	6 (13.0)	61 (13.9)	91 (15.0)	25 (13.2)	14 (14.0)	130 (14.5)
IPSS (documented by investigator)												
*n* with data	139	36	28	203	134	38	17	189	273	74	45	392
Low	20 (14.4)	2 (5.6)	5 (17.9)	27 (13.3)	29 (21.6)	3 (7.9)	2 (11.8)	34 (18.0)	49 (17.9)	5 (6.8)	7 (15.6)	61 (15.6)
Int‐1	38 (27.3)	8 (22.2)	6 (21.4)	52 (25.6)	34 (25.4)	15 (39.5)	4 (23.5)	53 (28.0)	72 (26.4)	23 (31.1)	10 (22.2)	105 (26.8)
Int‐2	49 (35.3)	17 (47.2)	12 (42.9)	78 (38.4)	40 (29.9)	17 (44.7)	8 (47.1)	65 (34.4)	89 (32.6)	34 (45.9)	20 (44.4)	143 (36.5)
High	32 (23.0)	9 (25.0)	5 (17.9)	46 (22.7)	31 (23.1)	3 (7.9)	3 (17.6)	37 (19.6)	63 (23.1)	12 (16.2)	8 (17.8)	83 (21.2)
IPSS (calculated from source data)												
*n* with data	73	28	16	117	45	10	6	61	118	38	22	178
Low	6 (8.2)	1 (3.6)	3 (18.8)	10 (8.5)	3 (6.7)	1 (10.0)	1 (16.7)	5 (8.2)	9 (7.6)	2 (5.3)	4 (18.2)	15 (8.4)
Int‐1	22 (30.1)	10 (35.7)	3 (18.8)	35 (29.9)	8 (17.8)	2 (20.0)	2 (33.3)	12 (19.7)	30 (25.4)	12 (31.6)	5 (22.7)	47 (26.4)
Int‐2	25 (34.2)	11 (39.3)	4 (25.0)	40 (34.2)	15 (33.3)	3 (30.0)	1 (16.7)	19 (31.1)	40 (33.9)	14 (36.8)	5 (22.7)	59 (33.1)
High	20 (27.4)	6 (21.4)	6 (37.5)	32 (27.4)	19 (42.2)	4 (40.0)	2 (33.3)	25 (41.0)	39 (33.1)	10 (26.3)	8 (36.4)	57 (32.0)
Baseline mutations, *n* (%)^b^												
Physician‐determined												
Number with data	105	27	19	151	62	16	9	87	167	43	28	238
*JAK2 V617F*	78	24	12	114 (75.5)	41	10	5	56 (64.4)	119	34	17	170 (71.4)
*JAK2* exon 12	1	0	0	1 (0.7)	0	1	0	1 (1.2)	1	1	2	2 (0.8)
*MPL W515*	1	0	1	2 (1.3)	2	0	0	2 (2.3)	3	0	1	4 (1.7)
Other (unspecified)	16	3	4	23 (15.2)	15	4	1	20 (23.0)	31	7	5	43 (18.1)
Centrally determined (OncoScreen)												
Number with data	18	3	3	24	65	22	8	95	83	25	11	119
*CALR*	3	0	1	4 (16.7)	15	0	2	17 (17.9)	18	0	3	21 (17.7)
*ASXL1*	2	0	1	3 (12.5)	12	1	1	14 (14.7)	14	1	2	17 (14.3)
*EZH2*	0	0	0	0	2	0	0	2 (2.1)	2	0	0	2 (1.7)
*IDH1*	0	1	0	1 (4.2)	0	0	1	1 (1.1)	0	1	1	2 (1.7)
*IDH2*	0	0	0	0	1	1	0	2 (2.1)	1	1	0	2 (1.7)
*SRSF2*	1	0	0	1 (4.2)	1	0	1	2 (2.1)	2	0	1	3 (2.5)
Transfusion need												
*n* with data	317	97	53	467	307	95	47	449	624	192	100	916
None	146 (46.1)	60 (61.9)	27 (50.9)	233 (49.9)	68 (22.1)	18 (18.9)	6 (12.8)	92 (20.5)	214 (34.3)	78 (40.6)	33 (33.0)	325 (35.5)
Low	121 (38.2)	34 (35.1)	19 (35.8)	174 (37.3)	189 (61.6)	74 (77.9)	36 (76.6)	299 (66.6)	310 (49.7)	108 (56.3)	55 (55.0)	473 (51.6)
Moderate	41 (12.9)	3 (3.1)	5 (9.4)	49 (10.5)	47 (15.3)	3 (3.2)	4 (8.5)	54 (12.0)	88 (14.1)	6 (3.1)	9 (9.0)	103 (11.2)
Strong	9 (2.8)	0	2 (3.8)	11 (2.4)	3 (1.0)	0	1 (2.1)	4 (0.9)	12 (1.9)	0	3 (3.0)	15 (1.6)
Palpable spleen												
*n* with data	195	65	28	288	113	47	17	177	308	112	45	465
Yes	156 (80.0)	61 (93.8)	22 (78.6)	239 (83.0)	64 (56.6)	33 (70.2)	10 (58.8)	107 (60.5)	220 (71.4)	94 (83.9)	32 (71.1)	346 (74.4)
No	39 (20.0)	4 (6.2)	6 (21.4)	49 (17.0)	49 (43.4)	14 (29.8)	7 (41.2)	70 (39.5)	88 (28.6)	18 (16.1)	13 (28.9)	119 (25.6)
ECOG status												
*n* with data	306	94	55	455	292	97	44	433	598	191	99	888
0	97 (31.7)	24 (25.5)	16 (29.1)	137 (30.1)	125 (42.8)	48 (49.5)	18 (40.9)	191 (44.1)	222 (37.1)	72 (37.7)	34 (34.3)	328 (36.9)
1	167 (54.6)	47 (50.0)	29 (52.7)	243 (53.4)	147 (50.3)	42 (43.3)	21 (47.7)	210 (48.5)	314 (52.5)	89 (46.6)	50 (50.5)	453 (51.0)
2	37 (12.1)	22 (23.4)	9 (16.4)	68 (14.9)	19 (6.5)	6 (6.2)	4 (9.1)	29 (6.7)	56 (9.4)	28 (14.7)	13 (13.1)	97 (10.9)
3	5 (1.6)	1 (1.1)	1 (1.8)	7 (1.5)	1 (0.3)	1 (1.0)	1 (2.3)	3 (0.7)	6 (1.0)	2 (1.0)	2 (2.0)	10 (1.1)
4–5	0	0	0	0	0	0	0	0	0	0	0	0
Hematology, median (IQR)^c^												
Hemoglobin (g/dL)	ND	ND	ND	11.1 (9.4–13.1)	ND	ND	ND	10.3 (9.2–11.9)	ND	ND	ND	ND
Leukocytes (×10^9^/L)	ND	ND	ND	13.2 (7.9–22.2)	ND	ND	ND	9.3 (6.5–16.0)	ND	ND	ND	ND
Platelets (×10^9^/L)	ND	ND	ND	297 (159–525)	ND	ND	ND	242 (137–401)	ND	ND	ND	ND

Abbreviations: BMI, body mass index; ECOG, Eastern Cooperative Oncology Group; Int, intermediate; IPSS, International Prognostic Score System; IQR, interquartile range; MF, myelofibrosis; ND, not determined; PMF, primary MF; PPV‐MF, post‐polycythemia vera MF; RBC, red blood cells; RUX, ruxolitinib; SD, standard deviation.

^a^ Missing BMI data for 152 patients overall (Arm A 64; Arm B 88).

^b^ Determined within a year before baseline.

^c^ Missing hematology data: Arm A—hemoglobin 29, leukocytes 59, platelets 50. Arm B—hemoglobin 24, leukocytes 62, platelets 41.

The most common causes of initial MF diagnosis were abnormal lab results (61%), splenomegaly (45%), weakness (28%), and constitutional symptoms (22%). Median (IQR) time since a confirmed MF diagnosis at study entry was 21.7 (1.1–77.6) months in Arm A and 39.6 (8.1–98.0) months in Arm B. Patients in Arm B had a median (IQR) of 117 (65–274) days prior exposure to RUX treatment; the longest time spent on RUX before study entry was 2366 days (6.5 years) and the shortest was 16 days.

Median (IQR) time on study was 2.08 (0.71–3.01) years in Arm A and 2.88 (1.03–3.02) years in Arm B, with a higher proportion of completers in Arm B (52%) than in Arm A (43%). Overall, the lowest number of completers was in PMF (277/643; 43%) and the highest in PPV‐MF (120/198; 61%), with the same pattern observed in both study arms. The median (IQR) duration of follow‐up among non‐completers was 11.5 (5.0–19.1) months. The most common cause of non‐completion was death, which occurred in 17% of patients overall (165/943, 33% of the dropouts). The proportion of deaths was similar in both arms and comparable for PMF (19%) and PPV‐MF (15%) but lower for PET‐MF (12%). Two deaths in Arm B were subsequent to the emergence of acute myeloid leukemia (AML), and one death in Arm A was subsequent to primary disease progression without AML. AE‐related non‐completion was twice as common in Arm A than in Arm B (12% vs. 6%), but there were no obvious differences between arms for other causes of non‐completion. There were no clear associations between BL characteristics and study completion (Table S1). As mutational analysis was not mandatory in the study, results on this can be found in the supplements (Supporting Information Part S2). After BL, 11 patients had a documented stem cell transplantation, 5 in Arm A and 6 in Arm B.

The vast majority of patients who discontinued (Table S1), while having completed the 36 month observation period, did so because of an AE (Figure S1). The details of these reasons for discontinuation (“adverse events” or “other”) were not further assessed in the CRF.

### 3.2 Study Dosing

Starting doses of RUX were higher for the RUX‐naïve patients in Arm A than for the experienced patients in Arm B. The median (IQR) starting dose in Arm A was 30 (20–40) mg/day (typically representing a dose of 15 mg twice daily [BID]), with 65% of patients initiating at 30 or 40 mg/day. Starting dose in Arm A was significantly (*p* < 0.05) correlated with both BL platelets (Pearson's rho 0.278) and hemoglobin (Pearson's rho 0.190), although the correlation coefficients were relatively small. Median (IQR) platelet count (×10^9^ cells/L) at BL was 112 (77–312) for patients starting at 10 mg/day, 371 (207–604) for 20 mg/day, 242 (159–447) for 30 mg/day, and 483 (296–725) for 40 mg/day. Median (IQR) hemoglobin (g/dL) at BL was similar for patients starting at 10 (10.2 [8.9–12.1]) and 30 mg/day (10.5 [9.3–12.8]), but higher for 20 (12.7 [10.8–14.4]) and 40 mg/day (12.3 [10.3–13.7]).

For patients in Arm B, who were already receiving RUX treatment, the median (IQR) dose at study entry was 25 (20–30) mg/day, with 55% of patients receiving 20 or 30 mg/day, 20% receiving 5–10 mg/day, and only 16% receiving 40 or 50 mg/day. As with Arm A, there were weak but statistically significant correlations between RUX dose at study entry and both platelets (Pearson's rho 0.145) and hemoglobin (Pearson's rho 0.106), although the correlation coefficients for Arm B were lower than for Arm A.

Final dosing in the study was similar between the two arms, with a median (IQR) dose of 20 (10–30) mg/day in Arm A and 20 (15–30) mg/day in Arm B, with similar proportions receiving 10 (19%–20%), 20 (22%–23%), 30 (24%–25%), and 40 mg/day (15%–18%).

Data on platelet count and starting dose at BL were available for 900 patients, with the vast majority of patients exhibiting platelets above 200 × 10^9^/L (62%) or between 100 and 200 × 10^9^/L (24%). Strikingly, in these two groups, 72% and 40% of patients, respectively, were dosed at a lower initial dose than prespecified in the SmPC. Similar observations were made for the patient groups with lower platelet counts: patients with platelets between 75 and 100 × 10^9^/L and platelets between 50 and 75 × 10^9^/L, were underdosed in 54% and 13% of cases, respectively. Interestingly, on the other hand, a substantial fraction of patients was dosed at a higher dose than recommended by SmPC in these groups (30% and 32%).

### 3.3 Efficacy

**FIGURE 1:**
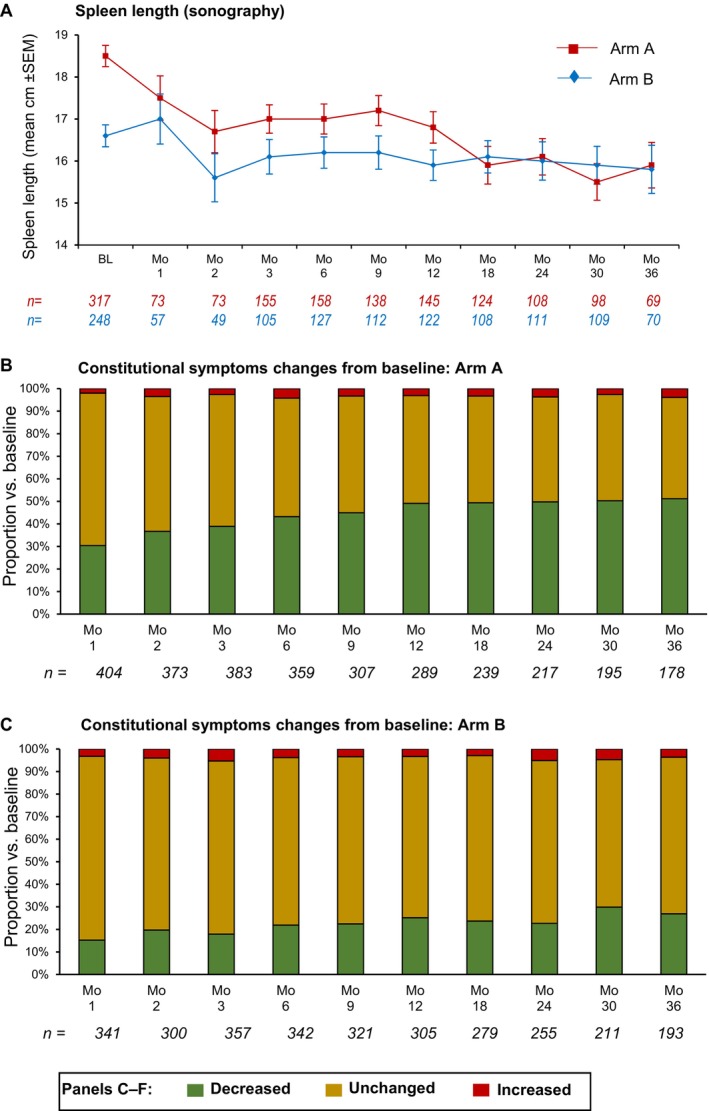
Study efficacy outcomes. (A) Mean spleen length over time; (B, C) proportions experiencing constitutional symptom changes from baseline in Arms A and B.

Mean BL spleen length by sonography was significantly larger in Arm A than in Arm B (Figure 1A), and, after a decline of ~2 cm over the first 2 months of treatment, this stabilized in arm A before declining further by ~1 cm at Month 12 and restabilizing between Months 18 and 36. Arm B mean spleen length dropped to a lesser extent (~1 cm) and then undulated around 0.5 cm below the BL level throughout Month 36. For the first 12 months of follow‐up, Arm B spleen length was consistently smaller than Arm A, but, following the second decline in Arm A, mean spleen lengths in both arms were essentially identical through to the end of follow‐up (Figure 1A).

Only a fraction of individual patients from Figure 1A had sonographic spleen length data from BL and at 12 months (111 patients in Arm A and 32 patients in Arm B). In Arm A, spleen length decreased from a mean of 19.0 cm to a mean of 16.89 cm, while in Arm B, spleen length decreased from a mean of 17.26 cm to a mean of 15.54 cm (standard deviations and medians do not corrupt reliability of these numbers). Compared to the data given in Figure 1A, it seems that the graphs in Figure 1A underestimate the treatment effect.

For Arm A only, changes in sonographic spleen length from BL to Month 6 were inversely correlated with the starting RUX dose (Pearson's rho −0.308; *p* < 0.05), and changes from BL to Months 6, 12, 24, and 36 were all correlated with the average RUX dose (Pearson's rho −0.184, −0.258, −0.254, and −0.362, respectively; all *p* < 0.05).

A similar ceiling effect on post‐BL improvement in Arm B was seen for the number of patients experiencing constitutional symptoms over time, for which the BL proportion in Arm A (65%) was also greater than in Arm B (39%). In Arm A, the proportion of patients with an improvement in constitutional symptoms was large and increased over the first 6–12 months of treatment, to then stabilize through to the end of follow‐up (Figure 1B), whereas, in Arm B, post‐BL improvement was much smaller and did not substantially change over the follow‐up period (Figure 1C).

Transfusion dependence (TD) is an important complication in MF patients and transfusion independence (TI) is a relevant treatment goal in these patients. In Arm A, among 254 TI patients at BL, 89 (36.3%) patients became TD, 156 (63.7%) patients remained TI. While, among the 234 patients TD at BL, 73 (31.2%) became TI and 161 (68.8%) remained TD. In Arm B, among 107 TI patients at BL, 20 (18.7%) patients became TD, and 183 (81.3%) patients remained TI. While, among the 357 patients TD at BL, 183 (51.3%) became TI and 174 (48.7%) remained TD.

In total, the following transfusion‐related AEs and SAEs were documented in this study: the numbers for transfusions representing AEs were 159 in Arm A and 111 in Arm B, respectively, and the number of transfusions representing SAEs were 26 in Arm A and 12 in Arm B, respectively.

### 3.4 Overall Survival

**FIGURE 2:**
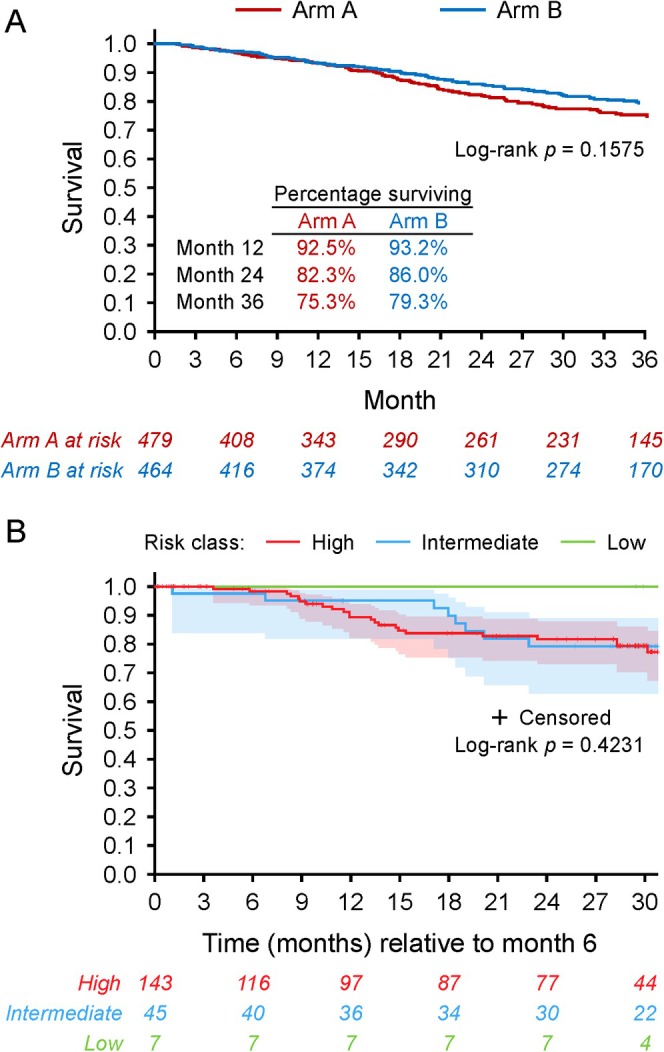
Overall survival by (A) study arm and (B) RR6 risk category in Arm A (sonographic spleen assessment only).

Overall survival was similar between Arms A and B, with at least three‐quarters of patients in each arm surviving through to the end of follow‐up, with no statistically significant difference between arms (Figure 2A).

On‐treatment risk stratification by the RR6 model was obtainable for a total of 256 patients in Arm A who had remained on treatment for at least 6 months; were documented through to subsequent discontinuation/death or study completion; and had relevant data at BL, Month 3, and Month 6. Of these, 195 were evaluated on the basis of sonographic spleen data in the primary analysis, and 61 were evaluated by palpation in the sensitivity assessment.

Of the 195 sonography patients, seven (4%) were classed as low RR6 risk, 45 (23%) as intermediate risk, and 143 (73%) as high risk. There were 31/195 (16%) post‐Month 6 deaths, of which, none occurred in the low risk stratum, eight occurred in the intermediate risk stratum (18% of the stratum), and 23 in the high risk stratum (16% of the stratum). There was no statistically significant difference in Kaplan–Meier survival between the Intermediate and high risk strata over the 30 months after Month 6 (Figure 2B), which is significantly different from the survival differences predicted in the original RR6 model publication [6].

### 3.5 AEs

With the exception of death, common, all‐cause, all‐grade AEs (≥ 5% overall incidence) and SAEs (≥ 2% overall incidence) are summarized in Table S2. The most common all‐grade AEs were RBC deficiencies (anemia 28%; hemoglobin reduced 9%), followed by thrombocytopenia (21%), and the most common individual SAE was pneumonia (6%). There were generally fewer individual AEs and SAEs among the RUX‐experienced patients in Arm B than in the previously naïve patients in Arm A, particularly with respect to anemia (24% vs. 32% for all‐grade AEs; 3% vs. 5% for SAEs), reduced hemoglobin (7% vs. 12% all‐grade AEs; 4% vs. 6% SAEs), and thrombocytopenia (12% vs. 29% all‐grade AEs; 2% vs. 3% SAEs). Most other common, all‐grade AEs and SAEs also showed numerically lower incidences in Arm B, with the exception of general physical health deterioration, iron overload, pain in extremities, and most common infections other than those of the urinary tract. Regarding non‐melanoma skin cancer (NMSC), a total of 13 AEs of basal cell carcinoma, 10 AEs of squamous cell carcinoma, 5 AEs of skin cancer, and 5 AEs of “skin neoplasm” were documented in the study. Only one patient was identified as diagnosed with a non‐Hodgkin lymphoma during follow‐up.

### 3.6 Patient‐Reported QOL Outcomes

**FIGURE 3:**
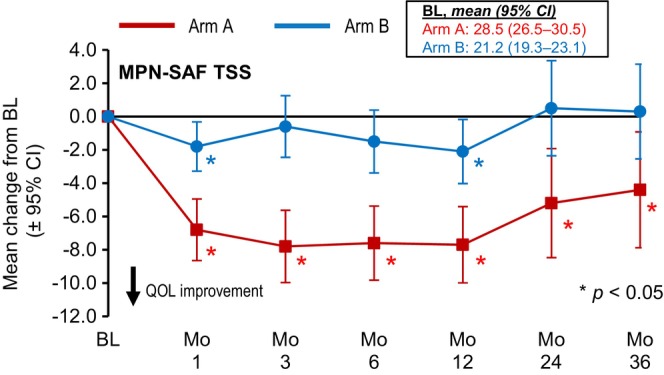
Changes from BL over time in patient‐reported outcomes. MPN‐SAF TSS.

The ceiling effects observed for efficacy parameters in Arm B were also apparent for patient‐reported QOL changes on study. Significant (*p* < 0.05) mean reductions from BL in the MPN‐SAF total symptom score (TSS), corresponding to an overall QOL improvement, were observed in Arm A at all assessment points, while Arm B scores remained essentially unchanged over the course of follow‐up (Figure 3). Notably, however, the Arm A mean BL TSS (28.5) was significantly higher than the Arm B BL TSS (21.2), and the maximum reduction in Arm A of ~7–8 points brought the Arm A TSS on treatment close to the BL for Arm B. Differences between subentities (meaning PMF/Post‐PV‐MF/Post‐ET‐MF separated by study arm) regarding improvement of TSS of 50% relative to BL have been included in the supplement (Table S3) showing numeric differences between MF subtypes over time.

The two component summary scores of the SF‐36 account for more than 80% of the reliable variance of the eight subscores [11]. An assessment was therefore undertaken in the proportion of patients in both arms experiencing a normal German QOL (score no lower than one SD below the overall German population mean) with respect to these summary scores at BL and throughout the follow‐up period. In Arm A, the proportion with a normal QOL with respect to the physical component summary score increased steadily from BL across the 36‐month follow‐up from 40.1% to 59.6%. By contrast, Arm B patients (already receiving RUX) entered follow‐up with a higher level of normal QOL (46.5%) that remained essentially unchanged over time (Figure S2A,B). A similar pattern was seen for the mental component summary score, where an early 9.5% increase in the proportion with a normal QOL from 54.5% at BL to 64.0% at month 12 was seen in Arm A, while Arm B entered follow‐up with a 63.8% level of normal QOL that remained essentially invariant (Figure S2C,D).

## 4 Discussion

JAKoMo was an observational, real‐world study reflecting routine clinical usage of RUX employed at individual physician's discretion for MF treatment in Germany. These real‐world data complement published clinical trial and expanded access results and reveal several features that differ from the clinical trial experience. Unfortunately, due to a limited data set and its focus on safety and efficacy, there was only limited data on patient details such as comorbidities collected and, therefore, further analysis, including their influence on outcomes may not be provided.

Interestingly, although Arm A starting doses of RUX were significantly and positively correlated with BL platelets as expected, the correlation coefficient was low (Pearson's rho 0.278), and the dosing distribution was lower than would be expected on the basis of the platelet distribution (based on recommendations specified in the European SmPC). Of note, these 30 and 40 mg/day strata were also used in the registrational COMFORT trials (which excluded patients with platelets below 100 × 10^9^/L). Median BL platelet count in JAKoMo Arm A was 297 × 10^9^/L (IQR 159–525 × 10^9^/L), which would put more than half the patients on the full 40 mg/day dose under SmPC prescription guidance, and the majority of the rest on 30 mg/day. However, only 27% of Arm A were prescribed 40 mg/day, while 39% received 30 mg/day and 19% received 10 mg/day. This reduced dosing intensity is surprising, especially since the RR6 prognostic model shows a dose of less than 40 mg/day over the first 6 months of treatment to be an established predictor of reduced MF survival [12]; however, this score was introduced much later. The lower starting dose and the weak but statistically significant correlation between starting dose and BL hemoglobin in Arm A (Pearson's rho 0.190) suggest that clinicians may have prescribed cautiously to minimize the potential for hematologic AEs, particularly in patients with lower hemoglobin. This lower dosing in the JAKOMO trial may reflect better knowledge about trials examining dosing schemes of ruxolitinib such as the phase 3b expanded access JUMP trial [6] mentioned above, and the REALISE Phase 2 Trial [13], in which patients were started at 10 mg bid and were eventually up‐titrated after 12 weeks of lower‐dosage treatment. However, as stated before, lower dosages did not lead to relevant reduction of anemia or infection but have been associated with reduced survival (e.g., RR6 prognostic model [12, 14]); therefore, physicians should strive to adhere to the dosing recommended in the SmPC. Dose reduction should be restricted to relevant clinical situations such as prohibitive adverse reactions or significant comorbidities.

Indeed, although differing approaches to AE ascertainment and grading complicate comparisons between JAKoMo and the COMFORT trials, there is some evidence that the conservative dosing in JAKoMo may, at least in part, have been associated with lower rates of some AEs. Comparing the reported incidence of clinically relevant post‐BL hematology changes in the JAKoMo Arm A with 3‐year follow‐up data for Grades 1–4 hematology events in the RUX arm of COMFORT‐II [15], fewer JAKoMo patients showed reduced levels of hemoglobin (32% vs. 82%), platelets (23% vs. 74%), neutrophils (6% vs. 16%), and leukocytes (11% vs. 23%). Over a median follow‐up of 112 weeks, JAKoMo documented lower rates of diarrhea (9% vs. 23%), nausea (7% vs. 13%), arthralgia (2% vs. 12%), nasopharyngitis (5% vs. 16%), and pain in extremities (4% vs. 12%) in Arm A compared with shorter‐term data from the primary 48‐week analysis of COMFORT‐II [4], despite the longer RUX exposure. On the other hand, there is nowadays a better knowledge of the potential side effects associated with ruxolitinib treatment and how to counteract or even prevent these AEs, possibly explaining the more favorable benefit–risk ratio in the JAKoMo trial.

Overall survival in JAKoMo was consistent with both the COMFORT studies, the expanded access JUMP study, and other real‐world analyses [14, 16]. Kaplan–Meier estimated survival for JAKoMo Arm A at Months 12 (93%) and 24 (82%) was consistent with JUMP overall survival estimates at Weeks 48 (94%) and 96 (87%) [6], while Month 36 survival in Arm A (75%) was comparable to Week 144 data for RUX patients in the pooled COMFORT studies (78%) [17]. Among the patients with available sonographic data, survival by RR6 risk category at Month 6 of RUX treatment did not clearly stratify over the course of follow‐up but was similar between Intermediate and High risk patients.

The relatively short (~4 months) median length of pre‐study RUX exposure in Arm B makes comparison to Arm A informative, despite a probable Arm B survivorship bias that would explain its higher completion rate and its lower rate of AE‐related discontinuations. Arm B patients entered the study with lower ECOG status, symptomatic burdens, hematology results, and spleen lengths, and, thereafter, showed little or no further improvement over the course of follow‐up for most outcomes other than spleen length. By contrast, patients in Arm A experienced immediate improvements in all these measures of disease activity.

Spleen length appeared to take longer to stabilize, with Arm B showing a small decline from study BL over the first 2–3 months, and Arm A showing a biphasic trajectory characterized by an initial decline over the first 2 months followed by stabilization and a second smaller and slower decline from month 9 to match Arm B spleen lengths from Month 18 onwards. Taken together, these data suggest that most clinical responses on RUX will reach a maximum improvement within the first 3–6 months of treatment, which will remain stable henceforth, while splenomegaly may show a two‐stage improvement, with a further decline in spleen size after approximately 1 year.

Significant and sustained improvement in the MPN‐SAF TSS was seen in JAKoMo Arm A from the first month of treatment to the end of follow‐up, which was reflected by significant improvements from BL to year 1 of treatment in most of the individual MPN‐SAF outcome measures. Improvements in the individual and summary scores of the SF‐36 were more variable and time dependent, but the largest and most sustained SF‐36 improvements were seen for vitality. The ceiling effect seen in Arm B for disease activity was also apparent in these QOL assessments, with Arm B patients entering follow‐up with better QOL scores and showing attenuated on‐study improvement versus Arm A for all measures. This might be reflection of ruxolitinib impacting physical and mental QoL mostly during the first months of treatment with persistence but no further improvement after several weeks of treatment.

In the real‐world setting of our study, RUX treatment was recommended to follow dosing recommendations according to SmPC, however, this was up to investigator to follow this guidance. This approach led to certain limitations of our analysis as, due to the observatory character of the trial with limited on‐site monitoring of the data, we faced a higher amount of missing values, particularly regarding IPSS scoring and molecular genetics assessments than usual for interventional clinical trials. However, following this approach, the data collected truly reflects clinical routine outside of clinical trials.

In conclusion, the JAKoMo study shows that real‐world RUX treatment of MF promotes significant and sustained clinical and QOL benefits, including general health improvement and alleviation of MF‐associated fatigue. Maximum sustained responses were generally achieved within 6 months and were associated with fewer AEs than in randomized trials, which may reflect more conservative and personalized real‐world dosing.

## Author Contributions

Data were documented by each center into an electronic case report form (eCRF). S.K., S.I., M.K., and H.L.P. analyzed the data and wrote the manuscript. Twenty study centers participated in our trial, 16 of them treated patients and collected data. All coauthors reviewed and amended the manuscript and agreed to the final version of the manuscript.

## Ethics Statement

The study was approved by institutional review boards and independent ethics committees at each center and conducted in accordance with all local legal and regulatory requirements, as well as the general principles set forth in the International Ethical Guidelines for Biomedical Research Involving Human Patients, Guidelines for Good Clinical Practice, and the Declaration of Helsinki.

## Consent

All patients provided written informed consent at study inclusion.

## Conflicts of Interest

S. Koschmieder reports research funding from Novartis, Bristol‐Myers Squibb, AOP Pharma, Janssen, Geron; advisory board honoraria from Pfizer, Incyte, Ariad, Novartis, AOP Pharma, BMS, Celgene, Geron, Janssen, CTI, Roche, Baxalta, Sanofi, Sierra Oncology, GSK, Abbvie, PharmaEssentia, Protagonist, and MSD; patent for BET inhibitor at RWTH Aachen University; honoraria from Novartis, BMS, Celgene, Geron, Janssen, Pfizer, Incyte, Ariad, Shire, Roche, AOP Pharma, GSK, Abbvie, MPN Hub, iOMEDICO, Astra Zeneca, and MSD; and other financial support (e.g., travel support) from Alexion, Novartis, BMS, Incyte, Ariad, AOP Pharma, Baxalta, CTI, Pfizer, Sanofi, Celgene, Shire, Janssen, Geron, Abbvie, Karthos, Sierra Oncology, Imago Bioscience, GSK, Abbvie, iOMEDICO, Protagonist, and MSD. S. Isfort reports advisory board honoraria from Pfizer, Incyte, GSK, Silence Therapeutics and Novartis; honoraria from Novartis, GSK, AOP Orphan, Abbvie, BMS, Pfizer, Incyte, and other financial support (e.g., travel support) from AOP Orphan, Alexion, Janssen, Novartis, Pfizer, Mundipharma, Roche, and Hexal. M. Reiser reports consultancy fees from Amgen, Janssen, Novartis, Stemline, Abbvie, Milteny, and travel support from Beigene. M. Koenigsmann reports honoraria from Pfizer, Abbvie, Bristol‐Myers Squibb, and AstraZeneca; travel support from Abbvie, Lilly, and Gilead Sciences. B. Heinrich reports consultancy fees from AstraZeneca, Daiichi Sankyo, and Boehringer Ingelheim; research funding from AstraZeneca, Jansen, Lilly, Novartis, and Roche; speakers bureau fees from AstraZeneca and Daiichi; and advisory fees from Gilead, Roche, and AstraZeneca. E. von der Heyde reports consultancy fees and honoraria from Novartis, Bristol‐Myers Squibb, and AstraZeneca. H. Tesch reports research funding from Lilly, and honoraria and travel support from Lilly, Novartis, Roche, GSK, Seagan, Pfizer, Lilly, AstraZeneca, Daiichi, Exact Science, and Vifor. B. Gröschl reports provided services for Novartis. P. Bachhuber reports employment with Novartis and holds equity in Novartis. S. Großer reports employment with Novartis and holds equity in Novartis. M. Koehler reports consultancy fees from Novartis. H.L. Pahl reports consultancy, honoraria, and advisory fees from Novartis and AOP Pharma; research funding from Novartis. The other authors declare no conflicts of interest.

## Supporting information


**Data S1.** Supporting Information.

## Data Availability

The datasets generated and/or analyzed during the current study are not publicly available but are available from the corresponding author on reasonable request and with permission of the steering committee of the trial and the sponsor.
