# Supplementary material for: Final Results From a Large, Non‐Interventional, Phase 4 Study of Ruxolitinib for the Treatment of Myelofibrosis in Clinical Routine
Source: Eur J Haematol. 2025 Jul 6;115(4):380–90. doi: 10.1111/ejh.70005 (PMC12402862; doi:10.1111/ejh.70005)
Supplement: Supplementary file 1 — Data S1. Supporting Information. [file EJH-115-380-s001.docx]

Supplementary Data

**PART S1:**

**ADDITIONAL METHODS**

The JAKOMO trial was a two-arm, non-interventional, prospective, observational study that followed 943 patients with PMF, PPV-MF, or PET-MF (including JAKi-naïve [arm A] and JAKi [RUX]-experienced [arm B] patients) who were prescribed RUX by hematologists or oncologists across Germany from September 2012 until September 2022. The 122 participating centers included academic institutions, community hospitals, and office-based settings. The follow-up period was 36 months from a patient’s baseline (BL) visit. RUX treatment, initial dosing, dose adjustments, suspensions or discontinuations, visit schedules, and clinical documentation were all at investigator discretion and in accordance with local clinical standards of care, as guided by the European Summary of Product Characteristics (SmPC) for RUX.

The primary objectives were to assess the utilization of RUX, its efficacy and safety, and its effects on patient QOL in routine clinical practice. Efficacy was evaluated as changes in spleen size while on treatment, improvements in constitutional symptoms and/or Eastern Cooperative Oncology Group (ECOG) performance status, hematological parameters, and survival. Safety was defined as the incidence and prevalence of adverse events (AEs) and serious AEs while on treatment, irrespective of suspected causality.

QOL was evaluated using two validated patient-reported outcomes questionnaires: the Myeloproliferative Neoplasm Symptom Assessment Form (MPN-SAF) and the 36-Item Short Form Health Survey v1 (SF-36 v1). The MPN-SAF was administered at BL and at 1, 3, 6, 12, 24, and 36 months of follow-up. The SF-36 was administered at study BL and months 6, 12, 24, and 36 of follow-up. While in the MPN-SAF low scores correspond to improved patient QOL, these are denoted by higher scores in the SF-36.

**STATISTICAL DETAILS**

On-study overall survival was assessed by Kaplan–Meier analysis by study arm and, for a subset of patients in arm A only, according to risk stratification by the RR6 prognostic model of Maffioli et al.^11^ Patients included in the RR6 analysis comprised a subset of arm A, those patients who remained on study for at least 6 months and had valid data at BL, month 3, and month 6 for RUX dose received, transfusions received (within 3 months prior to study for the BL assessment), and spleen size. RR6 risk assessment for death after the first 6 months of RUX treatment was determined as previously described.^11^ Although spleen size in the RR6 model was originally assessed by manual palpation, previous interim analysis has shown that measuring spleen length by palpation can lead to overestimation of actual spleen size^16^ and due to limited data availability of spleen size measurements by palpation, sonographic imaging data were used in this analysis.

SF-36 data were normalized against the normal German population. JAKoMo patients were considered to show a normal QOL for a given parameter if their individual normalized score was no lower than 1 standard deviation (SD) below the population mean.

**PART S2:**

**ADDITIONAL RESULTS:**

## Mutation analysis

BL mutation data (study entry ±6 months) were assessed locally by the treating physician (*n* = 238) or, from 2015 onwards, centrally by OncoScreen as an optional service (*n* = 119). Local assessments identified *JAK2* or *MPL* driver mutations only, with the presence of other mutations recorded but not specified in the dataset. Central assessments evaluated the presence of *CALR* driver mutations as well as high molecular risk (HMR) mutations. Both datasets are summarized in Table 1. As expected, the most common driver mutation was *JAK2 V617F* (71% of the locally assessed dataset), followed by *CALR* (18% of the OncoScreen dataset), with low incidences of both *JAK2* exon 12 and *MPL* mutations. The most common HMR mutation occurred in *ASXL1* (18%), at similar incidences in both arms. Mutations in *EDH2*, *IDH1/2*, and/or *SRF2* occurred in only ~2% of the patients each, with no obvious differences between arms.

# **TABLE S1**. Demographics and baseline characteristics by study completion status

|  | **Arm A (*n* = 479)** | | **Arm B (*n* = 464)** | | **All patients (*N* = 943)** | |
| --- | --- | --- | --- | --- | --- | --- |
|  | **Completed**  **(*n* = 208)** | **Not completed**  **(*n* = 271)** | **Completed**  **(*n* = 242)** | **Not completed**  **(*n* = 222)** | **Completed**  **(*n* = 450)** | **Not completed**  **(*n* = 493)** |
| Age, mean (SD), [range], years | 67.7 (10.6)  [38–89] | 72.2 (10.3)  [32–95] | 68.0 (11.5)  [30–86] | 72.8 (10.7)  [23–92] | 67.9 (11.1)  [30–89] | 72.5 (10.5)  [23–95] |
| Sex, *n* (%)  Male  Female | 114 (54.8)  94 (45.2) | 140 (51.7)  131 (48.3) | 125 (51.7)  117 (48.3) | 128 (57.7)  94 (42.3) | 239 (53.1)  211 (46.9) | 268 (54.4)  225 (45.6) |
| BMI, mean (SD), [range], kg/m^2^ | 25.1 (4.4)  [17.0–39.1] | 25.1 (4.3)  [16.0–45.0] | 26.3 (4.6)  [14.5–45.5] | 24.8 (4.0)  [15.6–40.1] | 25.7 (4.5)  [14.5–45.5] | 25.0 (4.2)  [15.6–45.0] |
| Smoking status, *n* (%)  *n* with data  No  Yes  Ex-smoker | 199  151 (75.9)  14 (7.0)  34 (17.1) | 259  198 (76.4)  26 (10.0)  35 (13.5) | 230  184 (80.0)  16 (7.0)  30 (13.0) | 208  162 (77.9)  15 (7.2)  31 (14.9) | 429  335 (78.1)  30 (7.0)  64 (14.9) | 467  360 (77.1)  41 (8.8)  66 (14.1) |
| IPSS (documented by investigator), *n* (%)  *n* with data  Low  Int-1  Int-2  High | 77  12 (15.6)  20 (26.0)  31 (40.3)  14 (18.2) | 126  15 (11.9)  32 (25.4)  47 (37.3)  32 (25.4) | 101  24 (23.8)  30 (29.7)  31 (30.7)  16 (15.8) | 88  10 (11.4)  23 (26.1)  34 (38.6)  21 (23.9) | 178  36 (20.2)  50 (28.1)  62 (34.8)  30 (16.9) | 214  25 (11.7)  55 (25.7)  81 (37.9)  53 (24.8) |
| IPSS (calculated from source data), *n* (%)  *n* with data  Low  Int-1  Int-2  High | 50  5 (10.0)  17 (34.0)  17 (34.0)  11 (22.0) | 67  5 (7.5)  18 (26.9)  23 (34.3)  21 (31.3) | 26  5 (19.2)  6 (23.1)  8 (30.8)  7 (26.9) | 35  0  6 (17.1)  11 (31.4)  18 (51.4) | 76  10 (13.2)  23 (30.3)  25 (32.9)  18 (23.7) | 102  5 (4.9)  24 (23.5)  34 (33.3)  39 (38.2) |

BMI, body mass index; Int, intermediate; IPSS, International Prognostic Score System; SD, standard deviation.

## **TABLE S2** Summary of common AEs and SAEs, irrespective of causality

| Any grade ≥5% or SAE ≥2% in all patients | **Arm A**  **(*n* = 479)** | | **Arm B**  **(*n* = 464)** | | **All patients**  **(*N* = 943)** | |
| --- | --- | --- | --- | --- | --- | --- |
|  | **Any grade** | **SAE** | **Any grade** | **SAE** | **Any grade** | **SAE** |
| Any AE | 433 (90.4) | 254 (53.0) | 400 (86.2) | 242 (52.2) | 833 (88.3) | 496 (52.6) |
| Anemia | 155 (32.4) | 25 (5.2) | 112 (24.1) | 12 (2.6) | 267 (28.3) | 37 (3.9) |
| Thrombocytopenia | 139 (29.0) | 16 (3.3) | 57 (12.3) | 9 (1.9) | 196 (20.8) | 25 (2.7) |
| Fatigue | 66 (13.8) | 3 (0.6) | 48 (10.3) | 1 (0.2) | 114 (12.1) | 4 (0.4) |
| Asthenia | 62 (12.9) | 9 (1.9) | 39 (8.4) | 4 (0.9) | 101 (10.7) | 13 (1.4) |
| Splenomegaly | 50 (10.4) | 6 (1.3) | 44 (9.5) | 4 (0.9) | 94 (10.0) | 10 (1.1) |
| Hemoglobin decreased | 56 (11.7) | 30 (6.3) | 32 (6.9) | 17 (3.7) | 88 (9.3) | 47 (5.0) |
| Dizziness | 46 (9.6) | 4 (0.8) | 31 (6.7) | 2 (0.4) | 77 (8.2) | 6 (0.6) |
| Pneumonia | 38 (7.9) | 28 (5.8) | 37 (8.0) | 29 (6.3) | 75 (8.0) | 57 (6.0) |
| Dyspnea | 42 (8.8) | 15 (3.1) | 32 (6.9) | 9 (1.9) | 74 (7.8) | 24 (2.5) |
| General physical health deterioration | 37 (7.7) | 23 (4.8) | 36 (7.8) | 23 (5.0) | 73 (7.7) | 46 (4.9) |
| Diarrhea | 45 (9.4) | 11 (2.3) | 25 (5.4) | 3 (0.6) | 70 (7.4) | 14 (1.5) |
| Nausea | 32 (6.7) | 3 (0.6) | 26 (5.6) | 2 (0.4) | 58 (6.2) | 5 (0.5) |
| Urinary tract infection | 35 (7.3) | 17 (3.5) | 20 (4.3) | 3 (0.6) | 55 (5.8) | 20 (2.1) |
| Pyrexia | 32 (6.7) | 16 (3.3) | 21 (4.5) | 8 (1.7) | 53 (5.6) | 24 (2.5) |
| Iron overload | 25 (5.2) | 3 (0.6) | 27 (5.8) | 4 (0.9) | 52 (5.5) | 7 (0.7) |
| Headache | 33 (6.9) | 2 (0.4) | 19 (4.1) | 1 (0.2) | 52 (5.5) | 3 (0.3) |
| Pruritus | 34 (7.1) | 1 (0.2) | 18 (3.9) | – | 52 (5.5) | 1 (0.1) |
| Bone pain | 28 (5.8) | 1 (0.2) | 21 (4.5) | – | 49 (5.2) | 1 (0.1) |
| Nasopharyngitis | 25 (5.2) | – | 23 (5.0) | – | 48 (5.1) | – |
| Epistaxis | 32 (6.7) | 6 (1.3) | 14 (3.0) | 1 (0.2) | 46 (4.9) | 7 (0.7) |
| Night sweats | 29 (6.1) | – | 17 (3.7) | – | 46 (4.9) | – |
| Pain in extremity | 20 (4.2) | 2 (0.4) | 24 (5.2) | 2 (0.4) | 44 (4.7) | 4 (0.4) |
| Cardiac failure | 21 (4.4) | 19 (4.0) | 21 (4.5) | 16 (3.4) | 42 (4.5) | 35 (3.7) |
| Fall | 23 (4.8) | 16 (3.3) | 11 (2.4) | 5 (1.1) | 34 (3.6) | 21 (2.2) |
| Atrial fibrillation | 18 (3.8) | 13 (2.7) | 13 (2.8) | 8 (1.7) | 31 (3.3) | 21 (2.2) |
| Sepsis | 16 (3.3) | 16 (3.3) | 13 (2.8) | 13 (2.8) | 29 (3.1) | 29 (3.1) |
| Pulmonary embolism | 14 (2.9) | 13 (2.7) | 6 (1.3) | 6 (1.3) | 20 (2.1) | 19 (2.0) |

Data are *n* (%).
AE, adverse event; SAE; serious adverse event.

## **TABLE S3** Arm A and B: Improvement of 50% relative to baseline: MPN-SAF total symptom score

| Arm A | | **P-MF**  **(N=325)**  **n (%)** | **PPV-MF**  **(N=99)**  **n (%)** | **PET-MF**  **(N=55)**  **n (%)** | **Total**  **(N=479)**  **n (%)** | |
| --- | --- | --- | --- | --- | --- | --- |
| *FU Month 1* | | | |  | |  |
| *-missing-* | *45(21.5)* | | *14(23.0)* | *13(30.2)* | | *72(23.0)* |
| *yes* | *42(20.1)* | | *15(24.6)* | *7(16.3)* | | *64(20.4)* |
| *no* | *122(58.4)* | | *32(52.5)* | *23(53.5)* | | *177(56.5)* |
| *FU Month 3* | | | |  | |  |
| *-missing-* | *54(25.8)* | | *15(22.4)* | *9(24.3)* | | *78(24.9)* |
| *yes* | *43(20.6)* | | *19(28.4)* | *7(18.9)* | | *69(22.0)* |
| *no* | *112(53.6)* | | *33(49.3)* | *21(56.8)* | | *166(53.0)* |
| *FU Month 6* | | | |  | |  |
| *-missing-* | *42(23.9)* | | *19(27.9)* | *8(25.0)* | | *69(25.0)* |
| *yes* | *33(18.8)* | | *21(30.9)* | *4(12.5)* | | *58(21.0)* |
| *no* | *101(57.4)* | | *28(41.2)* | *20(62.5)* | | *149(54.0)* |
| *FU Month 12* | | | |  | |  |
| *-missing-* | *35(24.1)* | | *14(25.5)* | *6(22.2)* | | *55(24.2)* |
| *yes* | *25(17.2)* | | *19(34.5)* | *6(22.2)* | | *50(22.0)* |
| *no* | *85(58.6)* | | *22(40.0)* | *15(55.6)* | | *122(53.7)* |
| *FU Month 24* | | | |  | |  |
| *-missing-* | *24(24.5)* | | *9(22.0)* | *6(30.0)* | | *39(24.5)* |
| *yes* | *21(21.4)* | | *9(22.0)* | *1(5.0)* | | *31(19.5)* |
| *no* | *53(54.1)* | | *23(56.1)* | *13(65.0)* | | *89(56.0)* |
| *FU Month 36* | | | |  | |  |
| *-missing-* | *23(28.4)* | | *7(23.3)* | *4(28.6)* | | *34(27.2)* |
| *yes* | *19(23.5)* | | *7(23.3)* | *1(7.1)* | | *27(21.6)* |
| *no* | *39(48.1)* | | *16(53.3)* | *9(64.3)* | | *64(51.2)* |

| Arm B | **P-MF**  **(N=318)**  **n (%)** | **PPV-MF**  **(N=99)**  **n (%)** | **PET-MF**  **(N=47)**  **n (%)** | | **Total**  **(N=464)**  **n (%)** | |
| --- | --- | --- | --- | --- | --- | --- |
| *FU Month 1* |  |  | |  | |  |
| -missing- | 37(22.2) | 22(44.0) | | 4(20.0) | | 63(26.6) |
| yes | 18(10.8) | 3(6.0) | | 2(10.0) | | 23(9.7) |
| no | 112(67.1) | 25(50.0) | | 14(70.0) | | 151(63.7) |
| *FU Month 3* | | | |  | |  |
| -missing- | 56(33.3) | 16(33.3) | | 7(25.9) | | 79(32.5) |
| yes | 19(11.3) | 6(12.5) | | 3(11.1) | | 28(11.5) |
| no | 93(55.4) | 26(54.2) | | 17(63.0) | | 136(56.0) |
| *FU Month 6* | | | |  | |  |
| -missing- | 48(29.4) | 15(31.9) | | 7(28.0) | | 70(29.8) |
| yes | 27(16.6) | 7(14.9) | | 3(12.0) | | 37(15.7) |
| no | 88(54.0) | 25(53.2) | | 15(60.0) | | 128(54.5) |
| *FU Month 12* | | | |  | |  |
| -missing- | 43(27.6) | 15(33.3) | | 7(29.2) | | 65(28.9) |
| yes | 28(17.9) | 7(15.6) | | 2(8.3) | | 37(16.4) |
| no | 85(54.5) | 23(51.1) | | 15(62.5) | | 123(54.7) |
| *FU Month 24* | | | |  | |  |
| -missing- | 34(32.1) | 20(41.7) | | 6(35.3) | | 60(35.1) |
| yes | 16(15.1) | 6(12.5) | | 2(11.8) | | 24(14.0) |
| no | 56(52.8) | 22(45.8) | | 9(52.9) | | 87(50.9) |
| *FU Month 36* | | | |  | |  |
| -missing- | 20(25.3) | 11(37.9) | | 4(33.3) | | 35(29.2) |
| yes | 10(12.7) | 2(6.9) | | 3(25.0) | | 15(12.5) |
| no | 49(62.0) | 16(55.2) | | 5(41.7) | | 70(58.3) |

## **FIGURE S1** Study disposition.


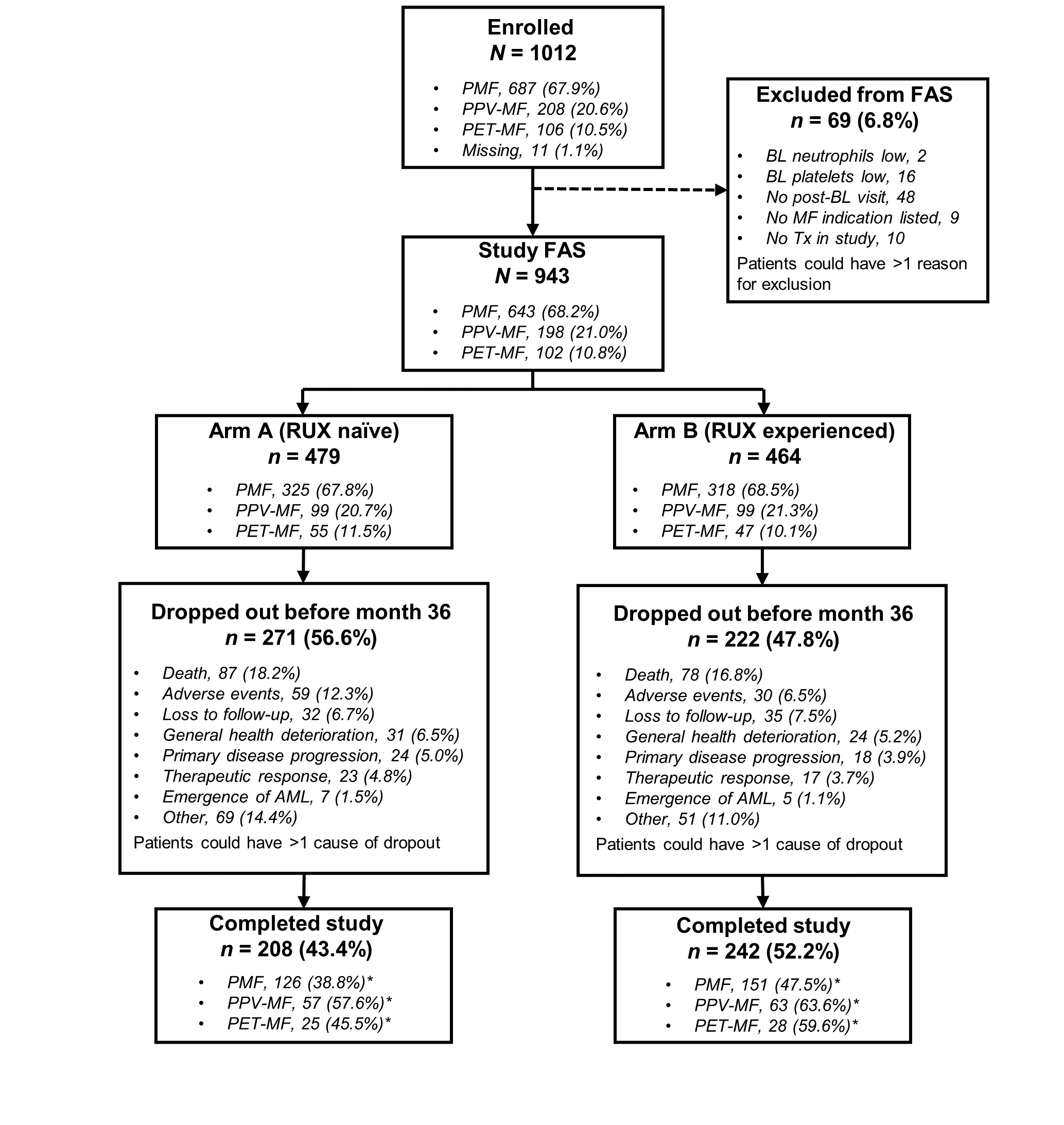


Percentage denominators are BL patients in the indicated study arm (A or B), except those marked “*”, which are BL numbers in the

indicated disease category (PMF, PPV-MF, PET-MF) within that study arm.

AML, acute myeloid leukemia; BL, baseline; FAS, full analysis set; MF, myelofibrosis; PET-MF, post-essential thrombocythemia MF;

PMF, primary MF; PPV-MF, post-polycythemia vera MF; RUX, ruxolitinib; Tx, treatment.

## **FIGURE S2** SF-36 v1 Z-score analysis. Absolute values and changes from BL in the proportions of patients on study experiencing a normal QOL with respect to the SF‑36 v1 physical component (A, B) and mental component (C, D) summary scores.


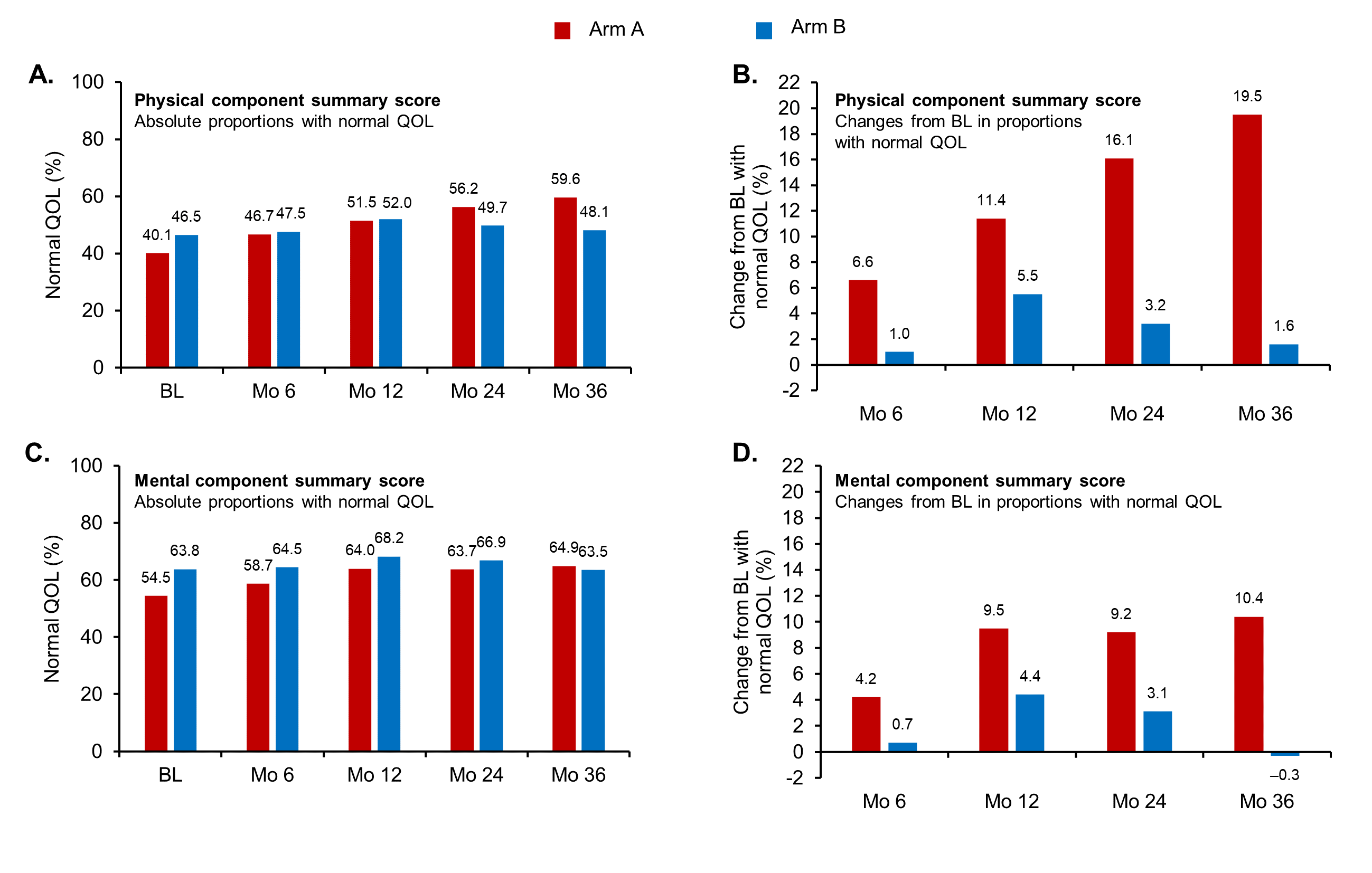


“Normal QOL” was defined as the proportion of patients with a normalized SF-36 v1 score no lower than 1 standard deviation below the mean of the overall German population.
BL, baseline; Mo, month; QOL, quality of life; SF-36 v1, 36-Item Short Form Health Survey version 1.
